# Sensitivity Enhancement in Low Cutoff Wavelength Long-Period Fiber Gratings by Cladding Diameter Reduction

**DOI:** 10.3390/s17092094

**Published:** 2017-09-13

**Authors:** Ignacio Del Villar, Matthew Partridge, Wenceslao Eduardo Rodriguez, Omar Fuentes, Abian Bentor Socorro, Silvia Diaz, Jesus Maria Corres, Stephen Wayne James, Ralph Peter Tatam

**Affiliations:** 1 Department of Electrical and Electronic Engineering, Public University of Navarra, 31006 Pamplona, Spain; ab.socorro@unavarra.es (A.B.S.); silvia.diaz@unavarra.es (S.D.); jmcorres@unavarra.es (J.M.C.); 2 Institute of Smart Cities, Public University of Navarra, 31006 Pamplona, Spain; 3 Engineering Photonics, School of Aerospace, Transport and Manufacturing, Cranfield University, Cranfield MK43 0AL, UK; m.c.partridge@cranfield.ac.uk (M.P.); s.w.james@cranfield.ac.uk (S.W.J.); r.p.tatam@cranfield.ac.uk (R.P.T.); 4 FIO Laboratory, Electronics Engineering Department, Universidad Autónoma de Tamaulipas, Tamaulipas CP 88740, Mexico; wrodriguez@docentes.uat.edu.mx; 5 Department of Telecommunications and Electronics, Pinar del Río University, Pinar del Río CP 20100, Cuba; omarf@upr.edu.cu

**Keywords:** fiber optics sensors, fiber bragg gratings, fibers, single-mode, etching, pH sensor

## Abstract

The diameter of long-period fiber gratings (LPFGs) fabricated in optical fibers with a low cutoff wavelength was be reduced by hydrofluoric acid etching, enhancing the sensitivity to refractive index by more than a factor of 3, to 2611 nm/refractive index unit in the range from 1.333 to 1.4278. The grating period selected for the LPFGs allowed access to the dispersion turning point at wavelengths close to the visible range of the optical spectrum, where optical equipment is less expensive. As an example of an application, a pH sensor based on the deposition of a polymeric coating was analyzed in two situations: with an LPFG without diameter reduction and with an LPFG with diameter reduction. Again, a sensitivity increase of a factor of near 3 was obtained, demonstrating the ability of this method to enhance the sensitivity of thin-film-coated LPFG chemical sensors.

## 1. Introduction

Long-period fiber gratings (LPFGs) have become increasingly popular in the domain of sensors, thanks to their principle of operation: the co-propagating coupling of light from the guided mode in the core of a single mode fiber to several modes guided in the cladding, which provides LPFGs with their sensitivity to strain, temperature, bending, and refractive index of the surrounding medium [1,2,3]. Changes induced in the effective index of cladding modes by perturbation of the refractive index of the surrounding medium lead to wavelength shifts of the attenuation bands created in the optical spectrum due to coupling between core and cladding modes, which has been exploited widely in the demonstration of chemical and bio-sensors [3]. 

In LPFG-based refractive index and chemical sensors, three phenomena can be combined with the aim of sensitivity enhancement (i.e., higher wavelength shifts as a function of the parameter to be detected): the mode transition [4,5,6], the dispersion turning point (DTP) [7,8], and cladding diameter reduction [9,10]. With an optimized design, sensitivities higher than 143,000 nm/refractive index unit (RIU) can be attained theoretically when considering the wavelength shift of the dual resonances created in the DTP region [11]. However, the practical implementation of this design is difficult and requires a high degree of accuracy in controlling the diameter of the optical fiber and the thickness of the nanocoating that must be deposited on the fiber. Despite these challenges, sensitivities of up to 40,000 nm/RIU for a single band in the DTP region have been attained experimentally [12].

In a recent publication, it was shown that, without the need for a nanocoating, it was possible to attain a sensitivity higher than 8000 nm/RIU by combining diameter reduction with operation at the DTP (considering the wavelength shift of the dual resonances created in the DTP region) [9].

In this work, we explore the use of an LPFG fabricated in a low cutoff wavelength optical fiber, which permits operation at shorter wavelengths, where the cost of light sources and optical spectrum analyzers is reduced significantly. Such a wavelength range is preferable for biosensing, one of the most challenging domains where optical fiber sensors are finding increased use.

## 2. Materials and Methods 

**Figure 1:**
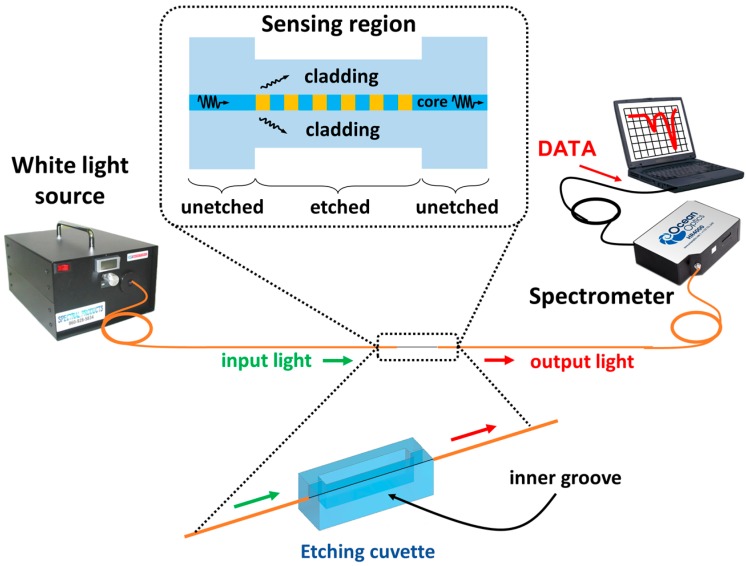
Experimental set-up. In the lower part, the cuvette used to etch the fibers is shown.

The experimental setup is depicted in Figure 1. Light from an ASBN-W tungsten-halogen broadband source from Spectral Products Inc. (Putnam, FL, USA) is launched into a boron-doped photosensitive optical fiber (Fibercore PS750, with a cutoff wavelength of 617 nm, a core diameter of 4.4 μm, a cladding diameter of 125 μm, and a numerical aperture of 0.12), and the output light is monitored by an HR4000 spectrometer (OceanOptics Inc., Largo, FL, USA). 

LPFGs with two different grating periods are analyzed: 109 μm and 118 μm. The reason for exploring two periods is that, according to [11], the period required to access the DTP depends upon the order of the cladding mode to which light is coupled, and, correspondingly, the wavelength at which the DTP is observed depends on the cladding mode order and thus the period. As will be shown later, in the optical fibers used in these experiments, an LPFG with a period of 109 μm allows coupling to the LP_0,19_ cladding mode at the DTP, while an LPFG with a period of 118 μm facilitates coupling to the LP_0,18_ mode at the DTP. This approach allows the comparison of the effect of etching LPFGs where the cladding mode coupled to at the DTP for the unetched fiber differs. 

The LPFGs are fabricated using the approach described in [13], where the fiber is illuminated, in a point-by-point fashion, by output from a frequency-quadrupled Nd:YAG laser operating at 266 nm, with a 7 ns pulse width, a rep-rate of 10 Hz, a beam diameter of 10 mm, and an average power of 200 mW. The output beam is conditioned using a focusing lens before being passed through a slit placed in front of the optical fiber. The fiber was kept straight during both the fabrication and the characterization processes to avoid any bending artifacts. It was found that a grating length of 3 cm produced deep resonance bands that were still visible in the spectrum after etching. Longer gratings were not used as they are more difficult to handle and more difficult to support if bending is to be avoided.

In order to etch the fibers, the cuvette depicted schematically in the lower part of Figure 1 is used. It consists of a horizontal squared plastic prism (HF dissolves silica but does not dissolve plastic) made in a 3D printer. The cuvette contains a 0.9-mm-wide groove where the fiber segment to etch can be placed without affecting the rest of the fiber and without releasing acid out of the cuvette. The design ensures that the fiber is kept straight, avoiding bend-induced distortion of the transmission spectrum.

In addition to this, some simulations were performed with FIMMWAVE in order to better understand the results obtained in the experiments. The grating used in the simulation consisted of a square wave to emulate the point-by-point technique used in the experimental inscription of the grating. The peak-to-peak modulation was 0.001. Modes LP_0,1_ up to LP_0,19_ were simulated, as, for the periods of the LPFGs used experiementally, LP_0,19_ is the higher-order cladding mode to which the core mode is coupled when the cladding diameter is 125 μm, i.e., before the fiber was etched etching. The finite difference method (FDM) was used, since it is the most accurate method available for a cylindrical waveguide. 

## 3. Results

**Figure 2:**
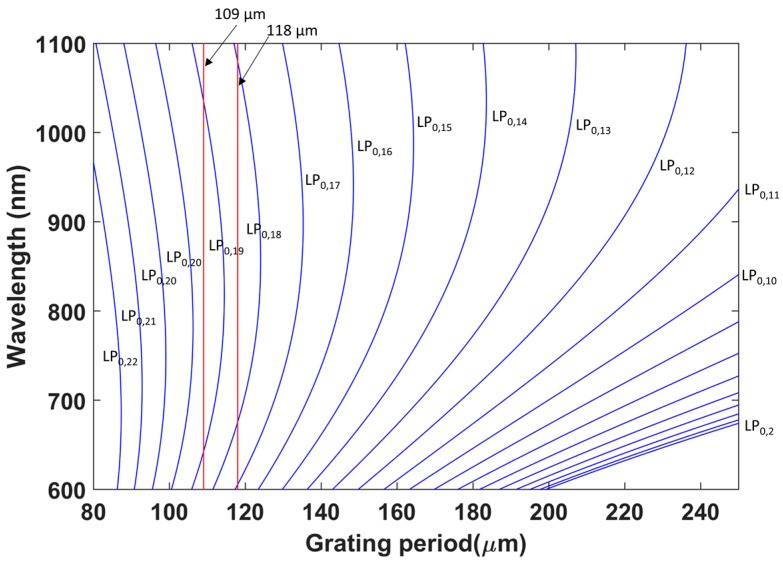
Calculated variation of resonance wavelength with grating period. For a period of 109 μm, the LP_0,19_ phase matching curve is intersected at two wavelengths, whereas for a period of 118 μm it is the LP_0,18_ phase matching curve that is intersected at two wavelengths. This indicates that for both periods the device operates close to a dispersion turning point (DTP).

As indicated in Section 2, LPFGs with two different periods were fabricated: 109 and 118 μm. The selection of these values can be better understood in Figure 2, where the resonance wavelength of each attenuation band is related to the grating period according to the well-known expression [3]:

(1) $$\lambda \;=\;\left[n_{co}\left(\lambda \right)\;-\;n_{clad}^{i}\left(\lambda \right)\right]\Lambda$$

where *n_co_* (*λ*) is the effective refractive index of the propagating core mode at wavelength *λ*, *n^i^_clad_* (*λ*) is the effective refractive index of the *i*th cladding mode, and Λ is the period of the LPFG.

Periods 109 and 118 μm intersect the phase matching curves at two wavelengths for each of the cladding modes LP_0,19_ and LP_0,18_ respectively. Thus, LPFGs operating at two different DTP orders were analyzed in this work.

**Figure 3:**
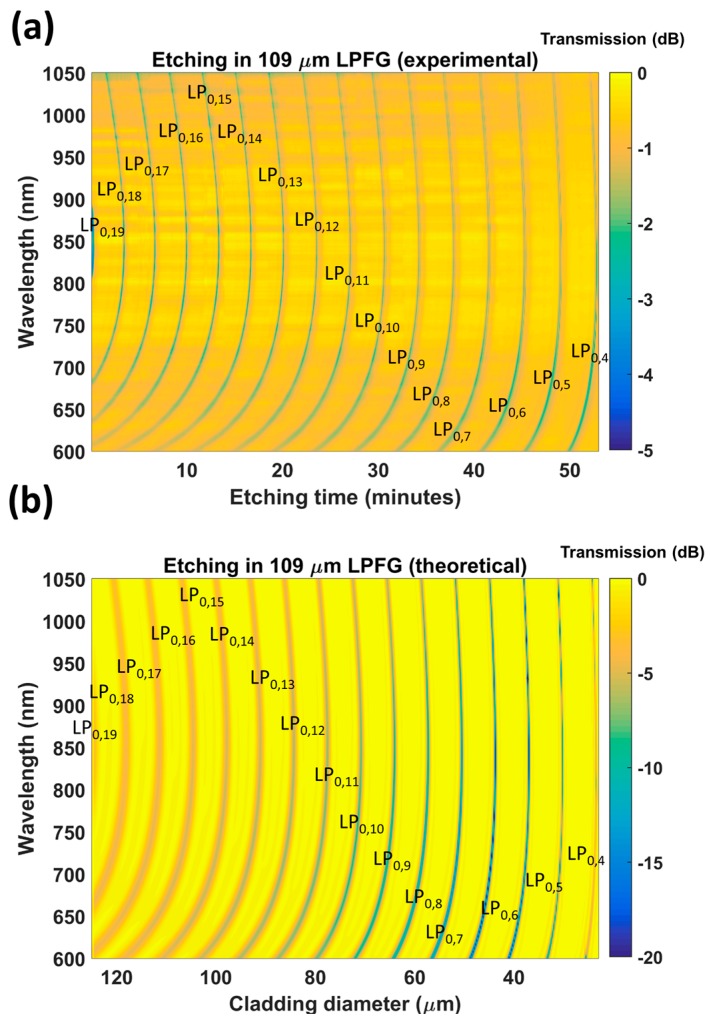
Evolution of the transmission spectrum of a long-period fiber grating (LPFG) with a period of 109 μm during etching: (**a**) experimental (the etching process lasted 53 min); (**b**) theoretical (based on the associated change in fiber diameter).

First, the section of fiber containing the 109 μm period LPFG was etched. Figure 3a shows a color map representing the transmitted power as a function of the wavelength and the etching time, whereas Figure 3b shows another color map with a simulation of the transmitted power as a function of the wavelength and cladding diameter. In both the experimental and theoretical results, the dark regions illustrate the shift of the dispersion turning points experienced by coupling to different cladding modes during etching (from LP_0,19_ down to LP_0,4_). The gradient increase in each band shows that the diameter reduction leads to a sensitivity increase. 

**Figure 4:**
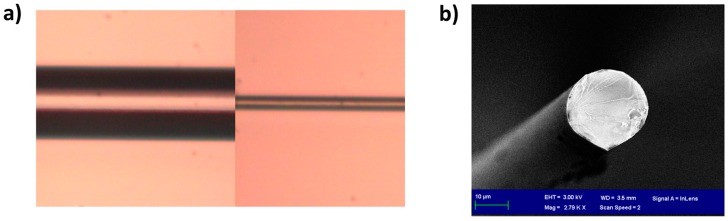
(**a**) Microscope image for a 125 μm fiber before and after etching. The diameter reduction factor is 5, which gives as a result a diameter of 25 μm; (**b**) cross section of an etched fiber. Reproduced with permission from: b Ref. [14], © 2017 IEEE.

According to the simulations, the diameter after etching was 24 μm. This value was obtained by relating the position of the LP_0,4_ band both in the experimental and in the theoretical results (Figure 3). In order to corroborate this value experimentally, another optical fiber was etched for 50 min and a similar value for the diameter of the fiber after etching was obtained: 25 μm (in Figure 4a, the ratio between the original fiber diameter of 125 μm and the diameter of the fiber after etching is 5).

Another important question is why the depth of the attenuation bands increases in the output from the simulation (Figure 3b), whereas this was not observed in the experimental data (Figure 3a). The reason can be found in the shape of the cross section of the etched optical fiber, which was also analyzed in Figure 10a of [14]. This image is reproduced in Figure 4b in order to illustrate the non-round shape of the optical fiber after etching. As a result of this asymmetry, coupling to the cladding modes is reduced in the experiments. This effect of reduction in the quality of the attenuation bands has also been observed in LPFGs operating in the telecommunications band [15]. A model for LPFGs with non-round cross sections could be developed to improve the similarity between experimental and theoretical results, but is beyond the scope of this work.

**Figure 5:**
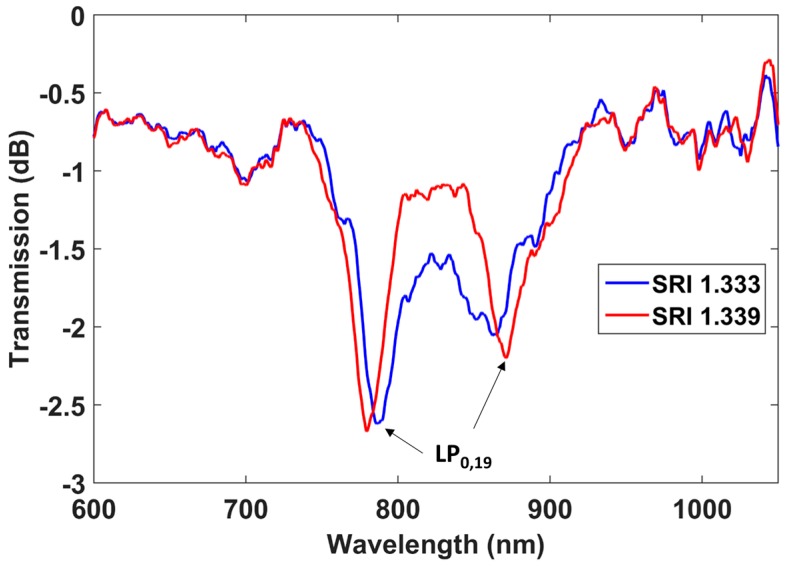
Transmission spectra of an LPFG of a period of 109 μm immersed in solutions of refractive indices 1.333 and 1.339 after etching.

The sensitivity of the LPFG after etching to surrounding refractive indices in the water region was determined by analyzing, as shown in Figure 5, the wavelength shifts of the attenuation bands in two different solutions: water (1.333) and glycerol in water (1.339). 

The wavelength shift of each resonance band was 8 nm. Consequently, the sensitivity of each band is 8/0.006 = 1333 nm/RIU. However, as was stated in the introduction, some authors define sensitivity as the ratio between two parameters: the increase or decrease of the separation between the left and the right band in the DTP region, and the surrounding refractive index variation [16,17]. Using this parameterization, the sensitivity is 2667 nm/RIU, which is larger than those obtained in DTP-tuned LPFGs in the telecommunications spectral range without cladding reduction (1309 nm/RIU in [16], 1847 nm/RIU in [17], and 944 nm/RIU in [18]), and is a third of the value of 8374 nm/RIU obtained with DTP-tuned LPFGs in the telecommunications spectral range with cladding reduction [9]. In all cases, the comparison has been performed in the water refractive index region, where many sensing applications take place. 

The lower sensitivity in the last case can be explained because the sensitivity is reduced at shorter wavelengths. It is a remarkable value considering the wavelength range where the device operates with the advantage of using less expensive optical sources and spectrum analyzers. Moreover, for these LPFGs, there is a higher number of bands within the spectrum, allowing the termination of the etching process at a higher number of different diameters, increasing the accuracy when controlling the sensitivity (the sensitivity depends on the fiber diameter).

**Figure 6:**
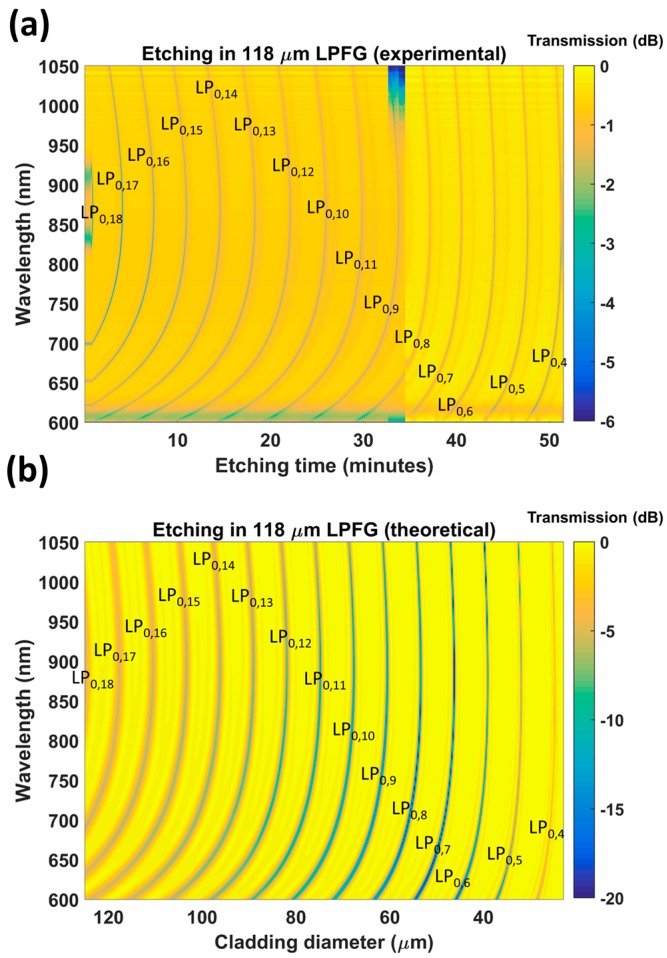
Evolution of the transmission spectrum of an LPFG with a period of 118 μm during etching: (**a**) experimental (the etching process lasted 51.5 min); (**b**) theoretical (based on the associated change in fiber diameter). Visualization 1 shows the evolution of the optical spectra during the etching process.

After analyzing the 109 μm period LPFG, the LPFG with a period of 118 μm was etched (see Figure 6). The initially observed DTP corresponded to a lower order mode: LP_0,18_. However, after etching for 51.5 min, a DTP was obtained that corresponds to coupling to LP_0,4_, the same cladding mode obtained with the 109 μm period LPFG. Visualization 1 shows the optical spectra as a function of time during the etching process.

By contrasting the simulations with the experimental results, it was possible to calculate the diameter of the optical fiber after etching: 24.7 μm, similar to the diameter obtained with the etching of the 109 μm LPFG. 

**Figure 7:**
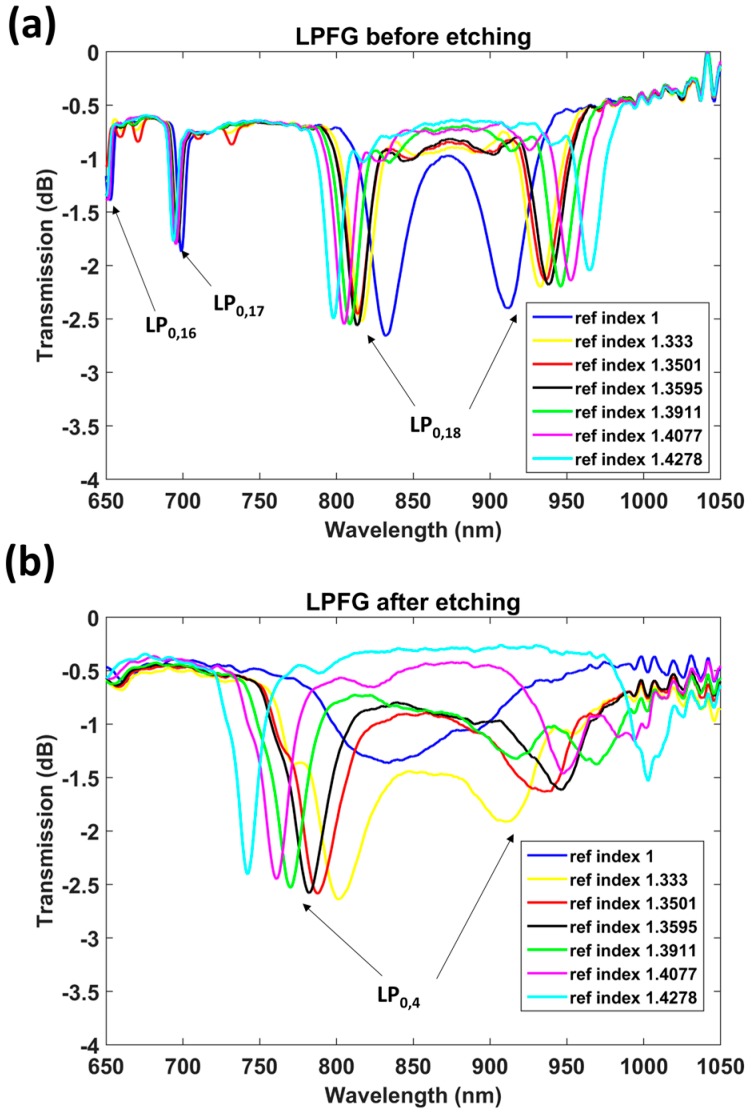
Transmission spectra for an LPFG of 118 μm immersed in different refractive indices after etching. (**a**) LPFG before etching; (**b**) LPFG after etching.

It is important to remark that the etching process in Figure 6a was performed in two steps, in order to prove if the process can be continued if the point of operation is not correctly tuned. In Figure 6a it is easy to observe that, at Minute 34, there is a change in the signal due to the extraction of the sensor from the cuvette and the further cleaning and immersion in the new HF solution. In Figure 7, the transmission spectra of the 118 μm period LPFG, recorded before and after etching, are presented. They correspond to immersion in air (refractive index 1) and in glycerol in water solutions with different refractive indices: 1.333, 1.3501, 1.3595, 1.3911, 1.4077, and 1.4278.

**Figure 8:**
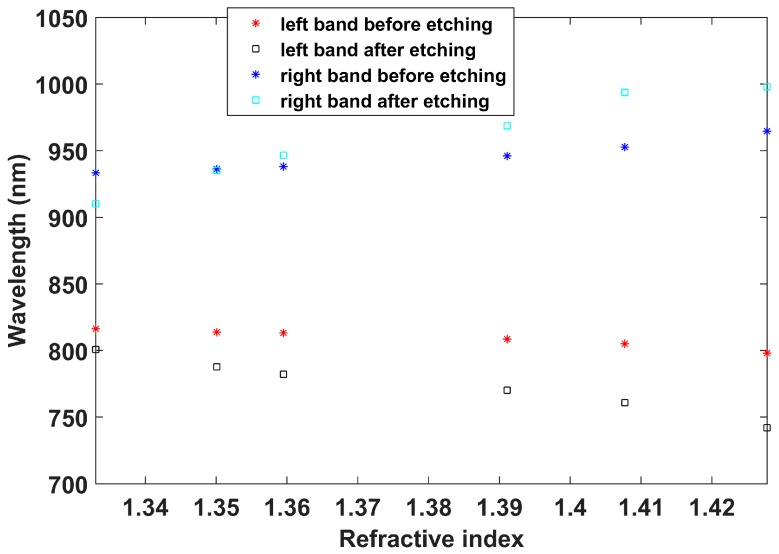
Wavelength shift of left and right attenuation bands before and after etching in a 118 μm LPFG.

In Figure 8, the wavelength shifts of left and right DTP bands before and after etching are compared. The sensitivity before etching was 319 nm/RIU in the left band and 508.9 nm/RIU in the right band, whereas the sensitivity after etching was 1058.7 nm/RIU in the left band and 1552.7 nm/RIU in the right band. Consequently, it can be said that the sensitivity was improved by a factor of more than 3 after etching, for an overall sensitivity of 2611.4 nm/RIU, by considering the separation of two bands. This value is similar to that obtained in Figure 5. However, the refractive index range analyzed(1.333–1.4278) was closer to the refractive index of silica than the range analyzed in Figure 5 (1.333–1.339), and it is well known that the sensitivity increases as the surrounding refractive index approaches that of silica. As a result, it can be stated that the sensitivity of the device characterized by Figure 5 is higher. The reason for this is that the attenuation bands are closer to each other (less than 100 nm in Figure 5 and more than 100 nm in Figure 7b). 

Another interesting observation is the position of the DTP, which in Figure 7b is centered at 850 nm, whereas in Figure 5 it is centered at 820 nm. The different wavelengths are a result of the different grating periods. The dispersion characteristics of the modes in the short cutoff wavelength fiber used here allows ready access to a number of DTPs, which is not the case for LPFGs operating at longer wavelengths, such as those analyzed in [11].

Finally, an application using etched LPFGs was developed. LPFGs with period 109 μm, one before etching and one after etching, were coated using the layer-by-layer assembly process to form pH sensors [19]. The coating consisted of 6 bilayers composed by alternating poly(allylamine hydrochloride) (PAH) and poly(acrylic acid) (PAA) polyelectrolytes, both of them in a 10 mM concentration and adjusted to pH 4.5 (purchased from Sigma-Aldrich, Saint Louis, MO, USA) [20].

**Figure 9:**
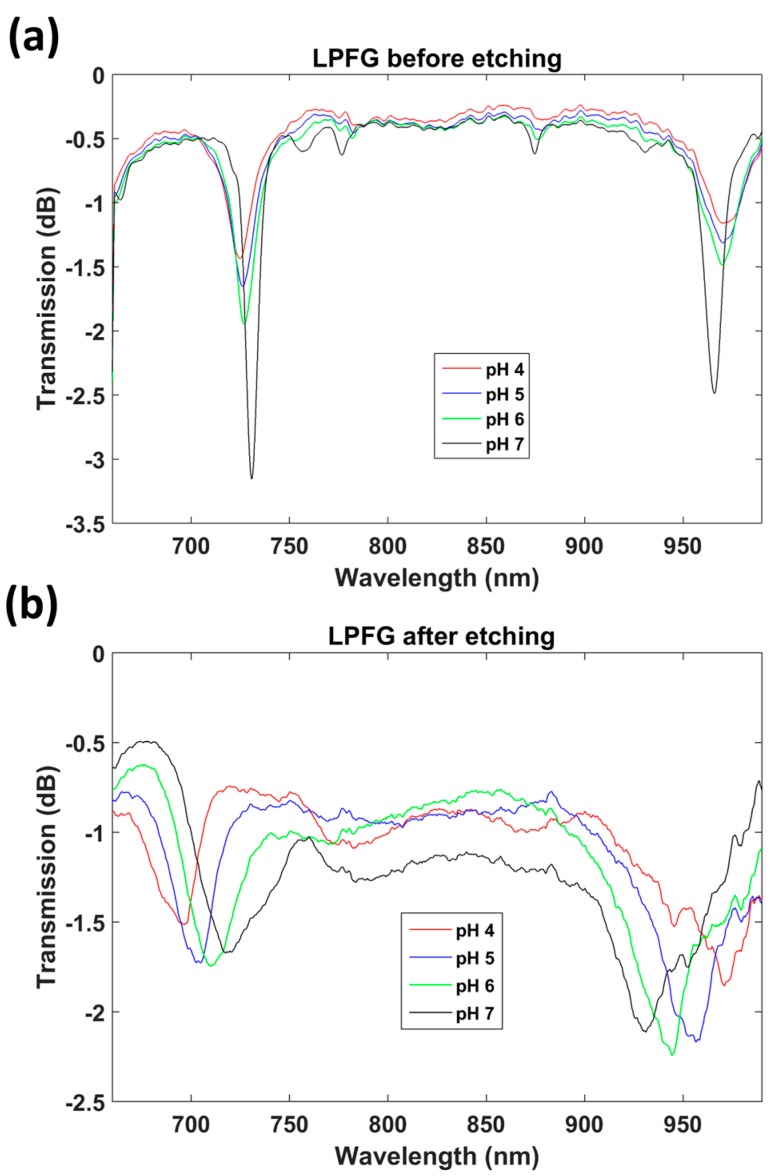
Transmission spectra of a poly(allylamine hydrochloride)/poly(acrylic acid) (PAH/PAA)-coated LPFG: (**a**) without cladding etching; (**b**) with cladding etching.

The decision to deposit the coating on the LPFG with a period of 109 μm rather than a period of 118 μm was based on two considerations. As was indicated above, the DTP resonance band is centered at a shorter wavelength for the LPFG with a period of 109 μm (820 nm as compared to 850 nm for the LPFG of 118 μm). According to [11], using a combination of grating period, etching, and deposition of a thin film, it is difficult to control the exact central wavelengths of the attenuation bands. Consequently, with a DTP centered at 850 nm, there are more possibilities that the longer wavelength band is shifted to wavelengths above 1000 nm, outside the range of the CCD spectrometer. Indeed, this is the case in Figure 9. Due to the fact that the DTP is centered at a shorter wavelength, it is possible to monitor the resonance band at 950 nm. A second reason for selecting a period of 109 μm is that, as was observed in Figure 6, one more DTP was observed during the etching process. Consequently, the number of diameters that can be selected for operation at a DTP is increased by one.

In Figure 9a, the transmission spectra of the unetched LPFG corresponding to four pH values are presented, and as shown in Figure 9b the same pH values were analyzed for the LPFG after etching. This time, the fiber was etched to allow access to the LP_0,10_ DTP. The reason is that, due to the combination of both the mode transition phenomenon and the diameter reduction, the sensitivity is so high that it is difficult to tune the position of the attenuation bands after etching to lie within the wavelength range monitored with the HR4000 spectrometer (Ocean Optics, Largo, FL, USA).

**Figure 10:**
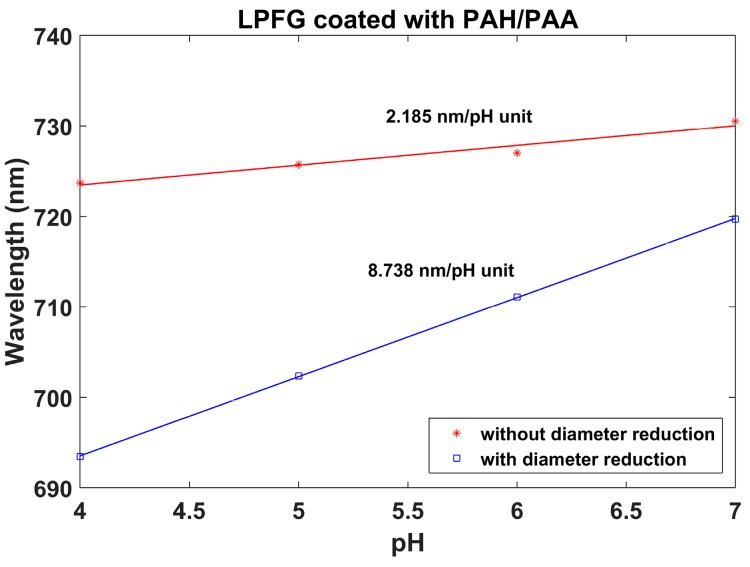
Wavelength shift of the left band for two PAH/PAA-coated LPFGs: one without clad etching and the other with clad etching.

In Figure 10, the pH-induced wavelength shifts of the attenuation bands of the etched and unetched sensors are compared with the lower wavelength band of the DTP. A MATLAB algorithm based on a moving average filter was used to calculate the position of the minimum at the wavelength range below 850 nm and the position of the minimum at the wavelength range above 850 nm. The value represented in Figure 10 is the average value during 5 min of immersion in each pH. The gradient of the wavelength shift for the LPFG without etching was 2.185 nm/pH unit, whereas, for the etched LPFG, a wavelength shift of 8.738 nm/pH was obtained. This is a 4-fold enhancement. It was also observed that the Q factor of the resonance bands is reduced after etching. It is not clear if this is a result of the reduction in diameter, or the effect of the deposition of the coating and proximity to the mode transition region, which has been shown previously to influence the Q factor [4,5,6]. Regarding the deposition of the thin film, the same coating thickness has been used for both the etched and unetched LPFGs, and the attenuation bands have similar central wavelengths. Consequently, the deposition of the thin film should affect the Q factor of the bands of both LPFGs equally. On the other hand, according to the simulations, high Q factors are also obtained for LPFGs with small diameters (in Figure 3b and Figure 6b, the resonances are very deep for small diameters). Therefore, the problem could be related to the non-circular cross section of the fiber, indicated in Figure 4b and discussed above. A rotating system that facilitates uniform etching could be a solution to this problem.

## 4. Discussion and Conclusions

The transmission spectra of low cutoff wavelength LPFGs were analyzed as a function of the cladding diameter. The numerical and experimental results show that the sensitivity to the refractive index of the surrounding medium increases as the cladding diameter decreases. At the same time, the sensitivity increases, as attenuation bands corresponding with coupling to different cladding modes are formed. Depending on the grating period, the initial attenuation band corresponds to a higher or lower order cladding mode. For instance, for an LPFG with a period of 109 μm, the initial band corresponded to the LP_0,19_ cladding mode, whereas for an LPFG with a period of 118 μm the initial band corresponded to LP_0,18_. However, after a hard etching of about 50 min, the attenuation band corresponding to the LP_0,4_ cladding mode was obtained, which according to the simulations corresponds with a cladding diameter of 25 μm. The conclusion is that, as the grating period is increased, the number of attenuation bands visible during the etching process is reduced. Hence, the number of points where it is possible to stop the process and to monitor a resonance for sensing purposes is reduced. This idea is reinforced when these results are compared with those obtained in [9], where with a grating period of 210 μm it was only possible to monitor 4 attenuation bands when etching from 80 to 32 μm, whereas here, for the 118 μm period LPFG, 16 attenuation bands could be monitored. The main conclusion is that shorter period gratings, suitable for attaining the dispersion turning point at short wavelengths, allow for the observation of many attenuation bands during the etching process, which facilitates the ability to stop the process at a specific diameter with the sensitivity the designer wants. Furthermore, optical sources and detectors are less expensive at shorter wavelengths, which is a positive in terms of manufacturing commercial devices.

In addition, even for the same cladding mode, it is possible to control the sensitivity of the device. For the 109 μm period LPFG, the etching processed was stopped in a point of high sensitivity in the water refractive index range: 2667 nm/RIU. This was possible because the two attenuation bands are very close to each other when the device is immersed in water. However, for the 118 μm LPFG, the bands were more separated from each other, and, even though the sensitivity in the range 1.33 to 1.4278 was 2611 nm/RIU, the refractive index range is closer to silica than in the other case (1.333 to 1.339), and it is well known that a higher sensitivity is attained as we approach the silica refractive index. It is important to mention that the sensitivity obtained in water doubles that of other works with LPFGs working at longer wavelengths, where the sensitivity of the device is inherently higher. 

Although there is still work to do in terms of improving the quality of the attenuation bands after etching (a system that guarantees a circular cross section of the fiber after etching is necessary), it has been shown that the etching process can be also be used to enhance the sensitivity when a nanocoating is subsequently deposited onto the fiber. As an example, a pH sensor for the range pH 4 to 7 was developed. The sensitivity increase after etching was more than a factor of 4, which opens the path towards the development of other applications such as chemical and biological sensors.

## Supplementary Materials

The following are available online at http://www.mdpi.com/1424-8220/17/9/2094/s1, Video S1: Visualization 1.
